# Acceptability of option B+ among HIV positive women receiving antenatal and postnatal care services in selected health centre’s in Lusaka

**DOI:** 10.1186/s12884-018-2142-1

**Published:** 2018-12-29

**Authors:** Bridget Chomba Chanda, Rosemary Ndonyo Likwa, Jessy Zgambo, Louis Tembo, Choolwe Jacobs

**Affiliations:** 1 Department of Paediatric and Child Health, University Teaching Hospital-HIV/AIDS Program, P.O Box, 50440 Lusaka, Zambia; 2 Department of population studies and Nutrition, School of Public Health, University of Zambia, Geneva, Switzerland; 3 Department of Epidemiology and Biostatistics, School of Public Health, University of Zambia, Lusaka, Zambia; 4 Department of Health Promotion and Education, School of Public Health, University of Zambia, Lilongwe, Malawi

**Keywords:** Acceptability, Antiretroviral therapy, Option B +, Prevention of mother to child transmission; knowledge, Attitude, Discrimination

## Abstract

**Background:**

In 2013, Zambia accepted the immediate operationalization of Option B+, a policy used to try and eliminate mother to child transmission. This policy requires all HIV-positive pregnant and breastfeeding women to initiate antiretroviral treatment for life regardless of CD4 count. However, not all HIV positive women accept treatment for life. This study aimed to investigate acceptability of lifelong ART (Option B+) among HIV positive women receiving antenatal and postnatal services at the university teaching hospital and Lusaka urban city clinics.

**Methods:**

This was a cross sectional study conducted in November, 2016 to March 2017. The study population comprised of HIV positive women in their reproductive age (15–49 years). A Structured questionnaire was used to collect data in a face to face interview with the participants. Data was entered in EpiData version 3.1 and analysed using Stata version 13. Multivariate logistic regression analysis was performed to determine predictors of acceptability.

**Results:**

Overall, 427 women participated in this study. Their mean age was 30 years. Of the 427, over half (54%) had inadequate knowledge and about 30% of the women in the study still experience stigma and discrimination.63.2% of the women had good attitude towards Option B+ and overall, the majority (77.8%) were willing to accept antiretroviral therapy for life. Multivariate analysis showed that only women with good attitude were 9.4 times more likely to accept Option B+ than those with a bad attitude [OR: 9.4: 95%CI, 5.8–15.2)].

**Conclusion:**

This study showed that in general, women accepted initiation of Option B+. However, there is still a gap in the level of knowledge of Option B+ as well as stigma and discrimination in some communities, hence there is need to intensify programs that are aimed at educating the community on the importance of ART for life, combat stigma and discrimination and consequently promote acceptability of Option B+.

## Background

About 160,000 new HIV infections among children aged 0–14 occurred in 2016, dramatically declining from 300,000 in 2010. Progress in reducing mother-to-child transmission of HIV has been dramatic since the introduction in 2011 of the ‘Global Plan towards the Elimination of New HIV Infections among Children, and Keeping their Mothers Alive’, largely because of increased access to PMTCT-related services and increased number of pregnant women living with HIV being initiated on lifelong antiretroviral medicines [1]. But it has not been fast enough to reach the 2020 targets set by UNAIDS and partners as part of the Super-Fast-Track Framework to end AIDS. Acceleration of treatment for all pregnant and breastfeeding women living with HIV is still needed to achieve elimination of new infections among children and halve HIV-related deaths among pregnant women and new mothers [1].

Under WHO’s 2010 PMTCT ARV guidance, countries had the option to choose between two prophylaxis regimens for pregnant women living with HIV with CD4 greater than 350cells/mm3. Option A and Option B. Under Option A, women received antenatal and intrapartum antiretroviral prophylaxis along with an antiretroviral postpartum “tail” regimen to reduce risk of drug resistance, while infants receive postpartum antiretroviral prophylaxis throughout the duration of breastfeeding [2].

Option B, on the other hand, has a simpler clinical flow in which all pregnant and lactating women with HIV initially are offered ART – beginning in the antenatal period and continuing throughout the duration of breastfeeding. At the end of breastfeeding those women who do not yet require ART for their own health would discontinue the prophylaxis and continue to monitor their CD4 count, eventually re-starting ART when the CD4 falls below 350cells/mm3. Along with these two options a third approach is now being used, Option B+, in which all pregnant women living with HIV are offered life-long ART, regardless of their CD4 count [2].

To achieve the goal of elimination of mother to child transmission (eMTCT), in 2013, the Zambian government accepted the immediate operationalization of Option B+ [3]. This is done with the understanding that all HIV positive women are to be initiated on antiretroviral therapy (ART) for life. Antiretroviral therapy offers women the best chance of preventing mother to child transmission, however, not all HIV positive women are willing to accept ART for life.

Katirayi et al. [4] found that women reported difficulty around learning their HIV status and initiating ART on the same day. They needed time to think about ART initiation and wanted to first discuss with their partners before committing to lifelong treatment. A study conducted in Zimbabwe found that pregnant and lactating women find it easy to accept lifelong therapy because it is similar to taking medication for diabetes or birth control [5].

Other studies have identified attitudes towards lifelong treatment [6], knowledge of prevention of mother to child transmission [7], social demographic [8] and cultural characteristics [9] as factors that determine acceptability of Option B+. However, the impact of these factors may vary depending on the study setting and study population.

Overall, the evidence regarding how women’s experiences in PMTCT option B+ services affect their subsequent care-seeking behaviour remains sparse. This appears particularly true with regard to the uptake of both long-term HIV care and treatment for the woman’s own HIV infection and infant HIV testing and related services. However, women’s experiences of and perspectives on current and proposed interventions and how these influence subsequent care-seeking behaviour need to be better understood to ensure that an appropriate and acceptable package of services could be offered and that the virtual elimination of mother-to-child HIV transmission become an attainable goal [10].

A study on acceptability of Option B+ among HIV positive pregnant and breastfeeding women is of supreme value in understanding the factors that inhibit enrolment on lifelong ART. During the implementation of different regimens for PMTCT, the efficacy of a regimen is not the only important aspect to consider but the way in which the people accept it will determine how well or how poorly it will work.

Study findings on the acceptability of Option B+ among Zambian women are limited. This study was conducted to determine acceptability of Option B+ and associated factors among HIV positive women receiving antenatal and postnatal care services at UTH and Lusaka Urban City Clinics. The information generated may be used to aid policy makers and HIV care givers in improving programmes aimed at encouraging the uptake of ART for life among pregnant and breastfeeding women and improve maternal health.

## Methods

### Study design and study setting

This was a cross sectional study conducted in five health centers in Lusaka from November, 2016 to March 2017. In Lusaka district, Maternal Child Health (MCH) and PMTCT services are offered in 24 of the 28 health centers and in all the health posts. The study sites (UTH, Matero Ref, Chawama, Kalingalinga and Chilenje) were chosen because they are in high density areas, offer antenatal, postnatal and PMTCT services.

### Sample size determination

A single population proportion formula was used to determine the study sample size with 95% confidence interval, 50% proportion, a precision of 5 and 10% non-response rate. The overall estimated sample size was 427. The study population comprised of HIV positive women who were attending antenatal and postnatal care services at the selected health centres and the target age group were women in the reproductive age (15–49). Exclusion criteria were HIV positive women with inability to communicate, HIV positive women who did not consent to take part in the study and all HIV negative women.

### Sampling method

This study used Stratified Random Sampling. Drawing from a total of 12 government owned delivery health facilities that reported their 2015 PMTCT annual program results to PEPFAR Zambia, five health facilities were selected. The chosen clinics and hospital were treated as strata’s. The number of women to be included in each health facility was worked out in proportion to the population of HIV positive women in each facility. Simple random sampling (SRS) was then used to select the study participants. The target population was selected from each health facility using a sampling list (PMTCT Register), taking into account the inclusion and exclusion criteria. The women were identified by their hospital numbers. These numbers were written on separate pieces of paper, folded and mixed into a box to conduct a lottery.

### Data collection and analysis

A structured interviewer administered questionnaire was used to collect data. Prior to administration of the questionnaire, a pilot study was conducted to check for appropriateness and consistency. The questionnaire was translated into Nyanja for those that did not understand or speak English. The questionnaire was divided into four sections namely, a) Socio-demographic factors, b) Knowledge of Option B+, c) Attitudes towards Option B+ and D) Socio-cultural factors.

Data was entered into EpiData version 3.1 and analyzed using Stata IC 13–64-bit (STATA Corporation, College Station, TX, USA) for analysis. Frequencies and proportions were used to describe the study participants. The statistical significance in this study was set at 5% (0.05) and confidence interval at 95%. Acceptability was the main outcome variable in this study and was binary. Independent variables associated with acceptability of Option B+ were analysed using logistic regression analysis. Univariate and multivariate logistic regression analysis were employed. The level of knowledge was measured from a score of five questions from the questionnaire, a score of four and five was graded as adequate knowledge and any score from 0 to 3 was graded as inadequate knowledge [6, 10]. Attitude was also measured using scores. A score of 3 was graded as good attitude and any score of 2 questions or less was graded as bad attitude [6, 10]. All variables found to be significantly associated with acceptability were included for multivariate logistic regression analysis to control for confounders.

### Ethics and consent

Ethical approval of the study was obtained from the University of Zambia Biomedical Research and Ethics Committee (IRB00001131 of IORG0000774, Reference number 022–06-16). Written consent was obtained from all the participants as well as parental consent for participants under the age of 16.

## Results

### Socio-demographic and cultural characteristics of participants

Table 1 shows the socio-demographic and cultural characteristics of the participants; most of them (48.7%) were within the age of 25–34 years with a mean age of 30. Majority (74.9%) were married at the time of the study and were not in formal employment. The educational levels of the respondents showed that 55.3% had reached secondary school while very few (17.1%) attained tertiary education. Regarding the socio-cultural factors, this study showed that most of the participants were Christian (98.6%) belonging to different Christian denominations. Thirty five percent (35%) of the participants expressed that their partners would discriminate them if they found out that they were on ART and most of them (68.6%) said they would not need permission from their partners or family members before initiating ART.

**Table 1:** Socio-demographic and cultural characteristics of participants

Demographic Factors		Frequency	Percent
Age	15–24 25–34 35–44 45–49	36 208 167 16	8.4 48.7 39.1 3.7
Marital status	single married divorced widowed	77 320 16 14	18 74.9 3.7 3.3
Occupation	employed unemployed	108 319	25.3 74.7
Education	primary secondary tertiary none	115 236 73 3	26.9 55.3 17.1 0.7
Cultural Factors			
Religion	Christianity Other	421 6	98.6 1.4
Denomination	Pentecostal Catholic Adventist	126 82 57	29.9 19.4 13.5
	UCZ Jehovah’s Witness other	51 28 78	12.1 6.6 18.5
Fear of partner discrimination	Yes No	152 275	35.6 64.4
Fear of family discrimination	Yes No	134 293	31.4 68.6
Permission to begin ART	Yes No	134 293	31.4 68.6
	Total	427	100

### Acceptability, level of knowledge and attitudes towards option B+

Table 2 shows that out of the 427 participants, only 45.9% had adequate knowledge of Option B+. Despite the level of knowledge found, most of them (63.2%) had good attitude towards Option B+ and overall, majority (77.8%) were willing to be on ART for life.

**Table 2:** Level of knowledge and attitudes towards Option B+ among participants

	Frequency	Percent
Knowledge level		
Adequate Knowledge	196	45.9
Inadequate knowledge	231	54.1
Specific attitudes towards Option B+		
Good attitude	270	63.2
Bad attitude	157	36.8
Overall Acceptability		
yes	332	77.8
no	95	22.2
Total	427	100

### Predictors of acceptability using logistic regression analysis

Table 3 shows the results of the logistic regression analysis. The univariate model showed that women who feared partner or family discrimination were less likely to accept Option B+ than those that believed that they would not be discriminated. Those with adequate knowledge were 2 times more likely to accept Option B+ than those with inadequate knowledge [OR: 2.1 (95%CI, 1.3–3.3)]. Attitude of women was observed to be statistically associated with acceptability with those with good attitude being 9 times more likely to accept Option B+ than those with a bad attitude [OR: 9.4 (95%CI, 5.8–15.2)].

**Table 3:** Logistic regression analysis on acceptability of Option B+ in relation to potential predictors

Predictor variable	COR (95% CI) *P*-Value		AOR (95% CI) *P*-Value	
Employment status				
Employed	1		–	–
Unemployed	0.8(0.5–1.4)	0.427		
Marital status				
Single	1			
Married	1.9(1.1–3.2)	0.028		
Divorced	2.1(0.5–8.0)	0.284		
Widowed	6.25(0.8–50.5)	0.086	1.0(0.6–1.7)	0.903
Education level				
None	1			
Primary	1.4 (0.1–16.2)	0.779		
Secondary	1.86(0.17–20.9)	0.615	–	–
Tertiary	2.1(0.18–24.9)	0.554		
Age				
> =35	1			
15–34	0.6(0.3–1.1)	0.099	–	–
Religion				
Other	1		–	–
Christianity	3.6(0.7–18.0)	0.122		
Christian Denomination				
Catholics	1		–	–
Protestants	1.1(0.6–2.0)	0.794		
Fear of partner discrimination				
No	1			
Yes	0.7(0.2–1.2)	0.003	1.0(0.5–2.0)	0.901
Fear of family discrimination				
No	1			
Yes	0.5(0.02–1.0)	0.041	0.7(0.4–1.4)	0.308
Permission to begin ART				
No	1		–	–
Yes	1.2(0.7–2.0)	0.481		
Knowledge				
Inadequate	1			
Adequate	2.1(1.3–3.3)	0.003	1.3(0.7–2.4)	0.356
Attitude				
Bad	1			
Good	9.4(5.8–15.2)	0.0001	9.3(5.7–15.2)	0.0001

All factors that were found to be significant in the univariate model were put in the multivariate model. The results show that attitude was the only factor significantly associated with acceptability of Option B+ [AOR: 9.2 (95%CI, 5.7–15.1)]. Participants with good attitude were 9 times more likely to accept Option B+ than those that had a bad attitude.

## Discussion

The majority of HIV positive women receiving antenatal and postnatal care services in this study accepted lifelong treatment. Similarly, studies from Malawi and Zimbabwe on the acceptability of Option B+ among pregnant and breastfeeding women showed that women accepted ART for life [4, 11].

While the women in this study generally accepted Option B+, there is still a gap in the knowledge levels. The study showed that over half (54.1%) of the participants had inadequate knowledge of Option B+. These findings were consistent with a study conducted in Tanzania which showed inadequate knowledge on treatment and prevention for HIV infected pregnant women: only 34% knew that HIV-infected pregnant woman could be on ART and 47% did not know that antiretroviral (ARVs) should be used throughout life [7]. A similar study from Ethiopia shows contradicting results, which was; most of the participants (58%) had adequate knowledge of PMTCT Option B+ [6]. The results of this study highlight a clear need for proving more information on Option B+ to the general population, women who attend antenatal and postnatal visits at health facilities as well as the benefits of being initiated on Option B+ early.

Regarding the socio-cultural factors, no association was observed between religion and denomination with willingness to accept option B+. The differences in the denominations among the women in this study did not affect acceptability. This is in line with a study that was conducted in Tanzania which showed that belief in the healing power of prayer was not significantly associated with a person’s hypothetical willingness to begin ARV treatment if they became HIV infected [12]. On the contrary, a study conducted in Ghana indicates that some respondents still held the opinion that HIV is a spiritual disease and therefore there is the need to seek spiritual interventions. Some defaulters cited “use of alternative medicines” as a reason for not going for antiretrovirals and others sleep in prayer camps. This causes them to default most often. However as disease progression among these groups becomes rapid they return to the health facility to continue treatment with ARVs [8].

This study also revealed that about 30% of the women still experience HIV related stigma and discrimination from their partners and families which may affect the acceptability of life-long treatment. Those who stated that their partner and family would discriminate them were less likely to accept Option B+ compared to those that believed they would not be discriminated. Research has shown that stigma and discrimination undermine HIV prevention efforts by making people afraid to seek HIV information, services and modalities to reduce their risk of infection and to adopt safer behaviour’s lest these actions raise suspicion about their HIV status [13].

With regards to getting permission from partner or family, most women in this study said they would not need any permission from their partners or family members before initiating ART for life. This implies that most women would not reject ART for life due to failure to get consent from partner/family. A study conducted in Malawi on Acceptability of lifelong treatment (Option B+) found contradicting results. The women were overwhelmed with the information, needed time to think about ART initiation and wanted to first discuss with their partners before committing to lifelong treatment [4].

Women in this study generally had a good attitude towards Option B+. Though receiving ART for life can be a burden; most of the women felt that they would not have problems with initiating ART for life. The good attitude found among the women could be attributed to the hope that ART can help improve the quality of life, survival and also benefit their children. This hope has helped change the negative attitudes towards lifelong treatment. Furthermore, the women with good attitude were more likely to accept Option B+ than the women with a bad attitude. This simply shows that the more the women develop a good attitude towards Option B+, the more the willingness to accept it. These findings were consistent with other research findings that have also shown that majority of the study participants 199 (66.1%) had positive attitude about PMTCT option B+ service [10]. A similar study conducted in Ethiopia reviewed that 76.3% of participants had positive attitude while others had poor attitude [6]. In order to have more HIV positive women on Option B+, intensive counselling needs to be done to help change the negative behaviours of some women.

However, this study has potential limitations that should be noted. Firstly this study was based on a cross-sectional design which does not show causal relationships. Secondly, Women who had been receiving ART prior to 2013 when Option B+ was implemented were included in this study. Generalization of the study findings was limited to selected health facilities of Lusaka District hence future studies should involve health facilities across the country.

## Conclusion

This study reveals that majority of the participants in this setting accepted the lifelong treatment of Option B+. Their attitudes towards it were generally good. However, we have found inadequacy on the level of knowledge of Option B+ which suggests need for efforts to help individuals and the broader communities have a better understanding of Option B+ as a preventive measure for HIV among women and their unborn children. Redesigning health educational strategies on PMTCT and ART for life into local language may reach both the literate and illiterate. There is also need to intensify sensitization programmes in order to successfully combat stigma and discrimination found in this study.

## Abbreviations

ART: Antiretroviral Therapy
ARV: Antiretroviral
EMTCT: Elimination of mother-to-child transmission (of HIV)
HIV: Human immunodeficiency virus
MTC: Mother to child transmission
PMTCT: Prevention of mother-to-child transmission of HIV
UNAIDS: Joint United Nations Program on HIV/AIDS
UNICEF: United Nations International Children’s Emergency Fund
UTH: University Teaching Hospital
WHO: World Health Organisation
